# Structural and Functional Implication of Natural Variants of Gαs

**DOI:** 10.3390/ijms24044064

**Published:** 2023-02-17

**Authors:** Yejin Jeong, Ka Young Chung

**Affiliations:** School of Pharmacy, Sungkyunkwan University, Suwon 16419, Republic of Korea

**Keywords:** G protein, GNAS, natural variants, conformation, GDP/GTP, inactivating PTH/PTHrP signaling disorders

## Abstract

Heterotrimeric guanine nucleotide-binding proteins (G proteins) are among the most important cellular signaling components, especially G protein-coupled receptors (GPCRs). G proteins comprise three subunits, Gα, Gβ, and Gγ. Gα is the key subunit, and its structural state regulates the active status of G proteins. Interaction of guanosine diphosphate (GDP) or guanosine triphosphate (GTP) with Gα switches G protein into basal or active states, respectively. Genetic alteration in Gα could be responsible for the development of various diseases due to its critical role in cell signaling. Specifically, loss-of-function mutations of Gαs are associated with parathyroid hormone-resistant syndrome such as inactivating parathyroid hormone/parathyroid hormone-related peptide (PTH/PTHrP) signaling disorders (iPPSDs), whereas gain-of-function mutations of Gαs are associated with McCune–Albright syndrome and tumor development. In the present study, we analyzed the structural and functional implications of natural variants of the Gαs subtype observed in iPPSDs. Although a few tested natural variants did not alter the structure and function of Gαs, others induced drastic conformational changes in Gαs, resulting in improper folding and aggregation of the proteins. Other natural variants induced only mild conformational changes but altered the GDP/GTP exchange kinetics. Therefore, the results shed light on the relationship between natural variants of Gα and iPPSDs.

## 1. Introduction

**Figure 1:**
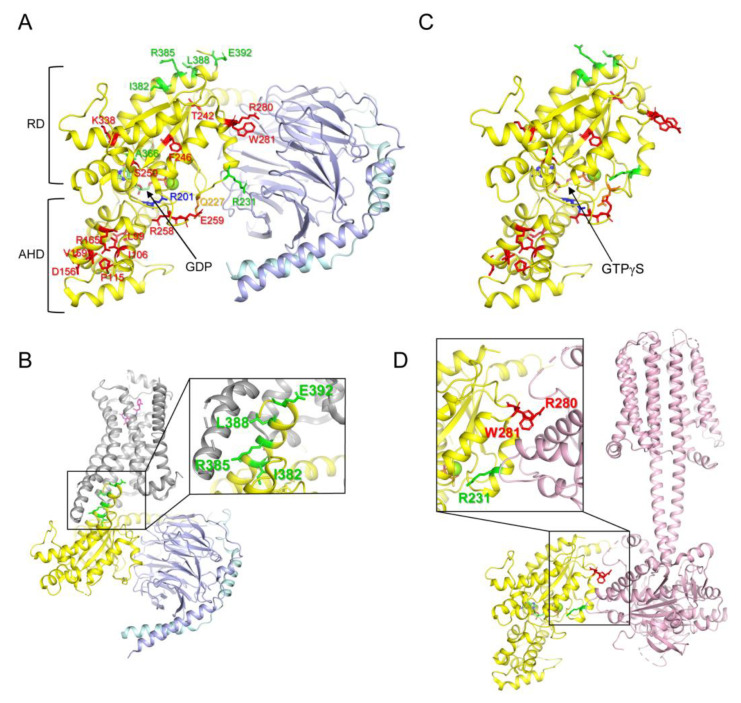
Structure of G proteins and mutated residues of the natural variants. (**A**) Structure of GDP-bound Gs (PDB: 6EG8) and reported mutated residues in the Gαs natural variants. Gαs, Gβ, and Gγ are colored yellow, light blue, and cyan, respectively. Mutated residues in the Gαs natural variants are shown as sticks. Red sticks present the residues analyzed in the current study; Green sticks present the residues whose molecular mechanism is well-understood; blue and orange residues are mutated in the gain-of-function natural variants. RD represents the Ras-like GTPase domain, and AHD represents the α-helical domain. (**B**) Structure of β_2_AR-Gs complex (PDB: 3SN6). Gαs, Gβ, Gγ, and β_2_AR are colored yellow, light blue, cyan, and grey, respectively. Mutated residues at the receptor-binding interface are shown as green sticks. (**C**) Structure of GTPγS-bound Gαs (PDB: 1AZT). Mutated residues in the Gαs natural variants are shown as sticks as described in (**A**). (**D**) Structure of the adenylyl cyclase-bound Gαs (PDB: 7PDE). Gαs and adenylyl cyclase 9 are colored yellow and light pink, respectively. Mutated residues at the adenylyl cyclase-binding interface are shown as green and red sticks.

Heterotrimeric G proteins (G proteins) are important cellular signaling proteins that are composed of Gα, Gβ, and Gγ subunits. Their activation is regulated by the binding of guanosine diphosphate (GDP) or guanosine triphosphate (GTP) to Gα [1]. In the basal state, Gα is bound to GDP and forms a heterotrimer with Gβγ (Figure 1A). The interaction of guanine nucleotide exchange factors (GEFs), such as G protein-coupled receptors, with Gα, induces the release of GDP (Figure 1B). Because of the higher concentration of GTP than GDP in the cytosol [2], Gα rapidly binds to GTP, which leads to conformational changes in Gα, resulting in the dissociation of Gα from the receptor and Gβγ. GTP-bound Gα (Figure 1C) is fully activated and regulates downstream signaling proteins, such as adenylate cyclase or phospholipase C [3,4,5,6,7,8]. The GTP bound to Gα is hydrolyzed into GDP due to the intrinsic GTPase activity of Gα, and Gα reassociates with Gβγ to form its basal state [1]. Gα consists of the following two domains: the Ras-like domain (RD) and the α-helical domain (AHD), and GDP or GTP is located between RD and AHD (Figure 1A,C) [9,10,11].

Based on the sequence and function of Gα, G proteins can be categorized into four subtypes (Gs, Gi/o, Gq/11, and G12/13) [12,13]. Each Gα subtype has distinct critical physiological functions in the cell; therefore, dysregulation of Gα leads to various diseases, such as tumors, cardiovascular diseases, neurodevelopmental disorders, and endocrine disorders [12,13]. Among the Gα subtypes, the misregulation of the Gαs gene (GNAS) is often driven by genetic alteration, and the relationship between genetic alteration and disease development has been extensively studied [14,15]. Gαs is ubiquitously expressed throughout the human body [12,13], and the most prominent function of GPCR-activated Gαs is the binding and activation of adenylyl cyclase (Figure 1D), which generates cAMP and activates protein kinase A [3,4,5]. Loss-of-function mutations in Gαs (Table 1) are often associated with parathyroid hormone-resistant syndrome such as inactivating parathyroid hormone/parathyroid hormone-related peptide (PTH/PTHrP) signaling disorders (iPPSDs) because parathyroid hormone receptors couple with Gαs [16]. Gain-of-function mutations in Gαs (Table 1) are often associated with McCune–Albright syndrome and tumor development [17,18,19].

The molecular mechanism underlying the gain-of-function mutations in Gαs is relatively well-defined; a gain-of-function mutation has been reported at Arg201 within switch I (Figure 1A, blue stick) and/or at Gln227 within switch II (Figure 1A, orange stick), and mutations at these sites reduce GTPase activity such that Gαs remains in the GTP-bound active state, leading to constant activation of Gαs [19,26,43].

Although a limited number of residues (i.e., Arg201 and Gln227) are reported to be altered in Gαs gain-of-function diseases, many residues throughout Gαs are altered in loss-of-function diseases (i.e., iPPSDs) (Figure 1A, red and green sticks) [20,21,22,23,30,33,34,35,36,37,38,39,40,41,42]. Among the natural variants of Gαs, the molecular mechanisms responsible for the loss of function have been described for only a few natural variants; for example, the alteration of the residues located at the receptor-binding interface (Figure 1B, green sticks, Ile382, Arg385, Leu388, and Glu392) disrupts the coupling with the receptors leading to parathyroid hormone resistance [40,41,42]; Arg231 is located at the Gβγ (Figure 1A) or adenylyl cyclase (Figure 1D) binding interface, and alteration of this site has been reported to interrupt the formation of the active structure [31,32,44]; and alteration of Ala366 reduces the affinity to GDP, leading to the destabilization of the conformation [39]. However, the precise functional consequences and molecular mechanisms of other natural variants of Gαs observed in iPPSDs (Figure 1A, red sticks) have not been extensively studied.

In the present study, we generated natural variants of Gαs that have not been extensively studied before (Figure 1A, red sticks) and analyzed the conformational and functional consequences of these natural variants. Conformational consequences were analyzed using hydrogen/deuterium exchange mass spectrometry (HDX-MS), and functional consequences were analyzed using BODIPY-labeled GTPγS (BODIPY-FL-GTPγS).

## 2. Results

### 2.1. Gαs Natural Variants That Lead to Aggregation in E. coli Purification System

We generated the natural variants listed in Table 1 (colored red) using PCR-based point mutation techniques and purified the natural variants as described in the Materials and Methods section. Several natural variants, namely L99P, F246S, S250R, R258W, E259V, and K338N, could not be purified because of aggregation of the expressed proteins, and the potential mechanisms of protein aggregation are discussed below.

**Figure 2:**
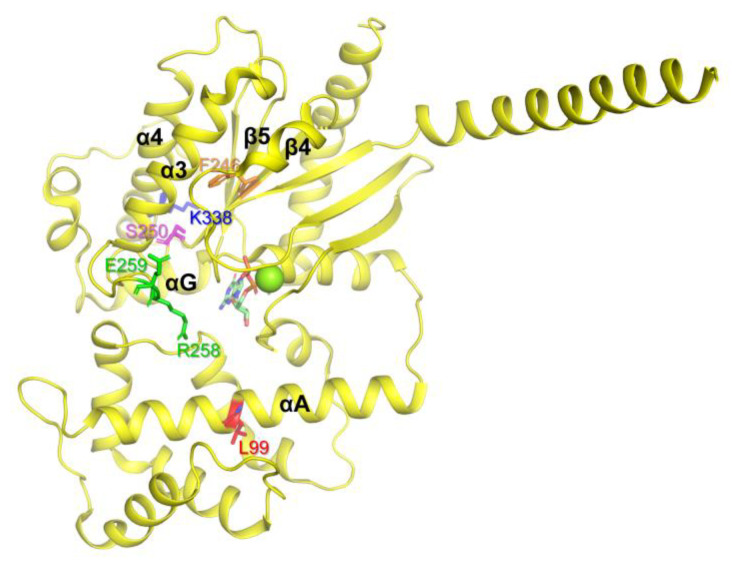
Gαs natural variants that underwent aggregation in the *E. coli* expression system. The Gαs natural variants that underwent aggregation are shown as sticks on the structure of GDP-bound Gαs (PDB: 6EG8).

The residue Leu99 is located in the αA region of AHD (Figure 2, red), and it has been suggested that αA determines the conformational stability of Gα. Because proline within the α-helix can bend the α-helix, the L99P variant could have produced significant conformational changes in αA, which destabilize the overall structure of Gα leading to aggregation. Phe246 is located in the β4 region, facing the inside of the protein (Figure 2, orange) and forming hydrophobic interactions. Thus, the switch from phenylalanine, which is hydrophobic and large, to serine, which is hydrophilic and small, could alter the overall conformational stability of the protein. Ser250 (Figure 2, pink) is located inside of the protein, and thus switching relatively small serin to relatively large and charged arginine would destabilize the overall conformation. Arg258 and Glu259 are located in Switch III (Figure 2, green). Switch III plays a critical role in GTP binding and interacts with the neighboring residues to form the proper conformation of the nucleotide-binding pocket. Natural variants in Switch III could have reduced GDP binding, resulting in the destabilization of the Gα structure leading to aggregation. Consistent with our speculation, previous studies showed that both R258W and E259V, when expressed in vitro transcription/translation technique, are more thermolabile than WT, and thus they denatured and aggregated at higher temperatures (30–37°) [35,45]. Lys338 is located in the α4 region (Figure 2, blue) and forms an ionic interaction with the neighboring residues within the αG/α4 loop. The αG/α4 loop is important for the interaction between RD and AHD, and we speculated that the structural alteration induced by K338N could affect both the conformational dynamics of the α4 helix and the RD/AHD interface, leading to structural instability. It should be noted that, in the current study, these natural variants were expressed in *E. coli*, and in the higher expression systems such as human cells, they might not be aggregated. However, the current study still suggests that these natural variants have lower conformational stability than the wild type, and thus are prone to be aggregated when the expression conditions are not optimal.

### 2.2. Natural Variants in AHD of Gαs

As shown in Figure 1A, Gα consists of RD and AHD, and GDP or GTP is present between them. RD is critical for canonical Gα functions, such as intrinsic GTPase activity and interaction with Gβγ (Figure 1A), receptors (Figure 1B), and downstream signaling proteins (Figure 1D) [1,10,11,13]. The functions of AHD have not been studied extensively, but it has been described as the regulator of the GDP/GTP turnover rate [46,47,48].

**Figure 3:**
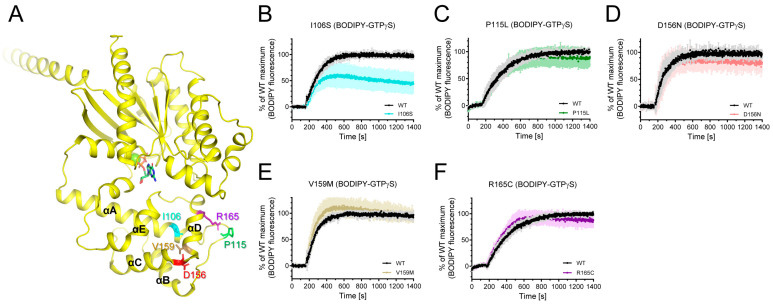
Effects of natural variants mutated within AHD on the intrinsic GDP/GTP turnover rate. (**A**) Positions of Gαs natural variants mutated within AHD are shown as sticks on the structure of GDP-bound Gαs (PDB: 6EG8). (**B**–**F**) BODIPY-FL-GTPγS fluorescence change of I106S (cyan) (**B**), P115L (green) (**C**), D156N (red) (**D**), V159M (brown) (**E**), and R165C (purple) (**F**). BODIPY-FL-GTPγS fluorescence was presented as percent (%) of WT Gαs maximum. Data are presented as the mean ± SD of more than three independent measurements.

Among the natural variants found in iPPSDs, a number are mutated within AHD (Figure 1A). Among these, the L99P variant was aggregated as described above, and we were able to purify I106S, P115L, V159M, D156N, and R165C variants (Figure 3A). We investigated whether these natural variants mutated in Gαs AHD affect the intrinsic GDP/GTP turnover rate using BODIPY-FL-GTPγS. BODIPY-FL-GTPγS has been successfully used to measure the relative GDP/GTP turnover rate [48,49]. The BODIPY fluorescence intensity of BODIPY-FL-GTPγS in solution is quenched by the neighboring guanine ring of GTPγS owing to the flexible bending of the linker. However, when BODIPY-FL-GTPγS binds to Gα, BODIPY is located away from the guanine ring, and the BODIPY fluorescence intensity increases. Therefore, the GDP/GTP turnover rate and maximum GTP incorporation can be analyzed by monitoring the fluorescence intensity.

The I106S variant altered the fluorescence intensity of BODIPY-FL-GTPγS (Figure 3B), whereas other natural variants mutated within AHD did not affect BODIPY-FL-GTPγS fluorescence (Figure 3C–F). The maximum fluorescence intensity of the BODIPY-FL-GTPγS with the I106S variant was lower than that with the wild-type (WT) Gαs (Figure 3B), suggesting that the maximum GTP incorporation is lower in the I106S variant than in the WT Gαs.

To further understand the conformational changes induced by the natural variants, we used HDX-MS. HDX-MS analysis elucidates the conformational dynamics of the protein backbone by monitoring the exchange rate between the hydrogen atoms within the peptide backbone amides and deuterium in the solvent [50]. We analyzed the HDX-MS profiles of GDP-bound WT Gαs and the natural variants. HDX-MS was performed as described in the Materials and Methods section, and the peptic peptides analyzed are summarized in Figure S1.

**Figure 4:**
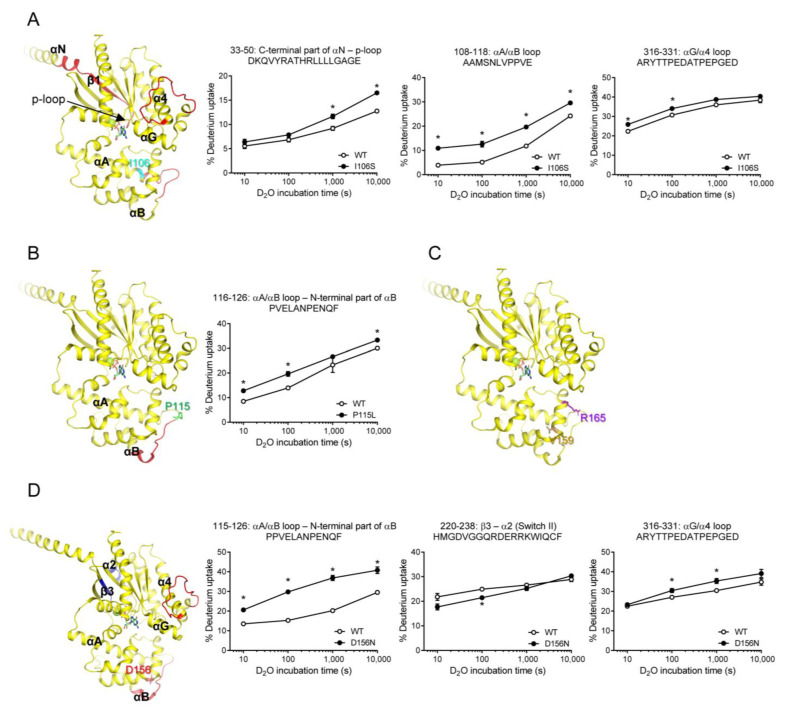
HDX-MS profiles of natural variants mutated within AHD. HDX-MS profiles of I106S (**A**), P115L (**B**), V159M and R165C (**C**), and D156N (**D**) are presented as color code on the structure of GDP-bound Gαs (PDB: 6EG8), and uptake plots of selected peptides are shown. Red represents higher deuterium uptake of Gαs natural variants compared to the WT. Blue represents lower deuterium uptake of Gαs natural variants compared to the WT. The mutated residues are shown as sticks. * indicates statistical differences between the natural variant and WT (*p* < 0.05).

The HDX-MS profile of the I106S variant differed from that of the WT Gαs in a few regions, including the C-terminal part of αN through the p-loop (peptides 33–50), αA/αB loop (peptides 108–118), and αG/α4 loop (peptides 316–331) (Figure 4A). Peptides 108–118 could be affected because they are present near the mutation site. Interestingly, the other two regions were far from the mutation site. Specifically, the p-loop is critical for nucleotide binding, and structural alterations in this region lead to altered GDP/GTP binding [51]. However, the αG/α4 loop was not directly involved in GDP or GTP binding. Thus, we speculated that the altered fluorescence intensity of the BODIPY-FL-GTPγS in the I106S variant (Figure 3B) was due to an altered conformation in the p-loop (Figure 4A, peptides 33–50).

P115L variant showed an altered HDX-MS profile near the mutation site (Figure 4B), but not in other regions, which could explain the lack of changes in the fluorescence intensity of BODIPY-FL-GTPγS (Figure 3A). Similarly, the V159M and R165C variants did not alter the HDX-MS profiles (Figure 4C,D), which is consistent with the results obtained for the fluorescence intensity of BODIPY-FL-GTPγS (Figure 3E,F).

The HDX-MS profiles of the D156N variant were different from those of the WT, not only near the mutation site (Figure 4E, peptides 115–126) but also in the regions far from the mutation site (Figure 4E, peptides 220–238, 316–331). The two regions affected in the RD were β3 through α2 and αG/α4 loops. Although β3 through α2 includes Switch II, which is important for nucleotide binding, the BODIPY-FL-GTPγS fluorescence intensity was not altered by the D156N variant (Figure 3D). This may be because the conformational change probed by HDX-MS (i.e., backbone amide hydrogen dynamics) does not affect nucleotide binding. In other words, if the conformational change proved by HDX-MS at β3 through α2 does not affect the coordination and binding of amino acid side chains with GDP or GTP, BODIPY-FL-GTPγS fluorescence intensity would not be altered by the D156N variant.

### 2.3. Gαs Natural Variants within RD

**Figure 5:**
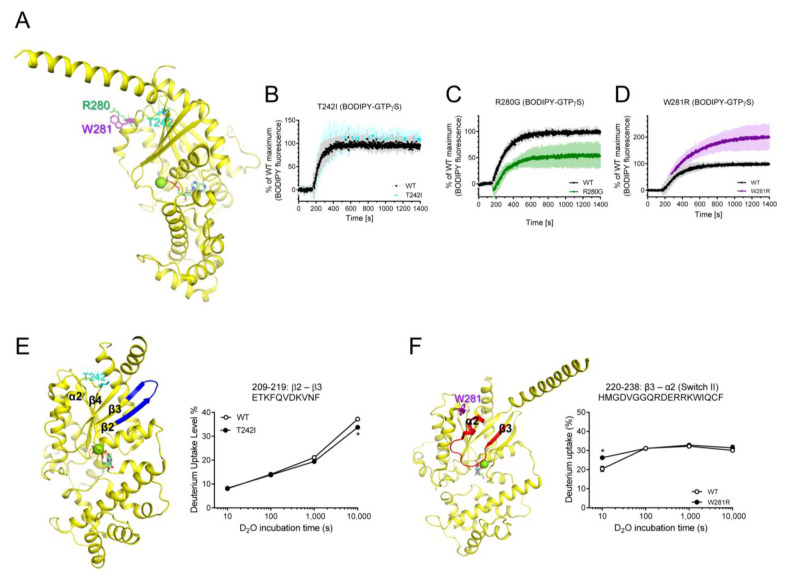
Effects of T242I, R280G, and W281R on the intrinsic GDP/GTP turnover rate and HDX-MS profiles. (**A**) Positions of T242I, R280G, and W281R are shown as sticks on the structure of GDP-bound Gαs (PDB: 6EG8). (**B**–**D**) BODIPY-FL-GTPγS fluorescence change of T242I (cyan) (**B**), R280G (green) (**C**), and W281R (purple) (**D**). BODIPY-FL-GTPγS fluorescence was presented as percent (%) of WT Gαs maximum. Data are presented as the mean ± SD of more than three independent measurements. (**E**,**F**) HDX-MS profiles of T242I (**E**) and W281R (**F**) are presented as color code on the structure of GDP-bound Gαs (PDB: 6EG8), and uptake plots of selected peptides are shown. Red represents higher deuterium uptake of Gαs natural variants compared to the WT. Blue represents lower deuterium uptake of Gαs natural variants compared to the WT. The mutated residues are shown as sticks. * indicates statistical differences between the natural variant and WT (*p* < 0.05).

Next, we tested the purifiable natural variants that are mutated within RD, T242I, R280G, and W281R (Figure 5A). The T242I variant was mutated in the α2/β4 loop, and it did not affect the fluorescence intensity of BODIPY-FL-GTPγS (Figure 5B), but the HDX-MS profile of β2 through β3 was slightly altered (Figure 5E). Interestingly, the two neighboring residues in the C-terminal of α3 showed opposite effects on the GDP/GTP binding when they are mutated (i.e., R280G and W281R) (Figure 5C,D). The R280G variant exhibited reduced maximum fluorescence intensity of BODIPY-FL-GTPγS (Figure 5C), although it did not affect the HDX-MS profiles. The W281R variant exhibited increased maximum GTP uptake (Figure 5D) and affected the conformational dynamics of Switch II (Figure 5F). Due to the lack of high-resolution structures of these natural variants, we do not know the structural mechanism that explains why R280G and W281R showed opposite GDP/GTP binding profiles. We speculate that the removal of the positively charged residues (R280G) or introduction of the positively charged residues (W281R) may differentially affect the allosteric conformational changes near the nucleotide-binding pocket through α3 or neighboring α2.

## 3. Discussion

In the present study, we analyzed the intrinsic GDP/GTP binding and conformational dynamics of natural variants of Gαs, which could elucidate whether these natural variants can cause iPPSDs. Several natural variants mutated in Gαs AHD, namely P115L, V159M, and R165C, did not affect the overall conformation (analyzed by HDX-MS) or GTP uptake (analyzed by BODIPY-FL-GTPγS fluorescence) (Figure 3 and Figure 4). These sites also are not involved in Gα functions such as receptor coupling or adenylyl cyclase binding. Therefore, we hypothesized that these natural variants would not cause iPPSDs.

However, the natural variants mutated in the αA region in AHD significantly affected the structure and function of Gαs; the L99P variant induced reduced conformational stability such that it aggregated when expressed in *E. coli*, and the I106S variant altered the conformation of the p-loop and reduced maximum GTP uptake (Figure 3B and Figure 4A). A previous study reported that the conformational dynamics of AHD could affect the GDP/GTP turnover rate. Dohlman et al. revealed the X-ray crystal structure of GPA1, a self-activating G protein α subunit of *Arabidopsis thaliana*, which suggested that the higher conformational fluctuation at αA of GPA1 than that of Gαi1 is related to the faster GDP/GTP turnover rate of GPA1 than of Gαi1 [46,47,48]. In a recent study, we reported that AHD is responsible for the different GDP/GTP turnover rates between Gαi and Gαo subtypes [51]. We further defined that the N-terminal part of αA is a regulator of the GDP/GTP turnover rate, whereas the C-terminal part of αA is not only a regulator of the GDP/GTP turnover rate but also of the maximum GTP binding [51]. Thus, significant structural alteration induced by the L99P variant might compromise GDP binding so that the protein becomes aggregated, resulting in the reduction of functional proteins within the cell. Moreover, conformational changes at the C-terminal part of αA by the I106S variant would reduce GTP binding as observed in Figure 3B, resulting in the reduction of receptor-mediated Gαs activation. Therefore, we suggest that the natural variants mutated in αA lead to loss-of-function forms of Gαs thus can cause iPPSDs.

D156N, another natural variant mutated in AHD, did not alter the fluorescence intensity of BODIPY-FL-GTPγS (Figure 3D), but it induced conformational changes at β3 through the α2 region (Figure 4E). Although this variant did not affect intrinsic GDP or GTP binding, since α2 is the major binding interface for Gβγ or adenylyl cyclase (illustrated in Figure 1A,D), D165N might affect the interaction with these proteins, resulting in loss of function, which requires further investigation.

Among the tested natural variants mutated in RD, F246S, S250R, R258W, E259V, and K338N induced protein aggregation when expressed in *E. coli* potentially due to reduced conformational stability, which would lead to the loss of functional proteins in the cell; therefore, these natural variants can cause iPPSDs.

Other natural variants mutated in RD showed altered intrinsic GDP/GTP binding and/or altered conformational dynamics. The R280G variant exhibited reduced GTP binding, which may lead to lower receptor-mediated Gα activation (Figure 5C). Therefore, we suggest that the R280G variant could cause iPPSDs. Surprisingly, the W281R variant exhibited increased intrinsic GTP uptake compared to that of the WT (Figure 5D), which could lead to increased receptor-mediated Gαs activation. However, Trp281 faces Gβγ (Figure 1A) or adenylyl cyclase (Figure 1D), and W281R altered the structure of the α2 region (Figure 5F), which may affect the binding of Gβγ or adenylyl cyclase. Whether the W281R variant alters interaction with Gβγ or effector proteins should be further investigated to determine whether W281R can cause iPPSDs. Lastly, T242I did not alter GTP uptake (Figure 5B) but slightly altered the structure of β2 through the β3 region (Figure 5E), which is important for receptor binding. Therefore, whether T242I alters receptor binding should be investigated.

In summary, this study reveals that natural variants L99P, I106S, F246S, S250R, R258W, E259V, R280G, and K338N could induce iPPSDs by either introducing protein aggregation or reducing GTP binding. The HDX-MS analysis also suggested that the involvement of D156N, T242I, or W281R variants in iPPSDs development should be investigated by examining the interaction of these natural variants with Gβγ, adenylyl cyclase, or receptors, as these natural variants altered structure at the switch II and/or receptor binding interface. We also suggest that natural variants P115L, V159M, and R165C would not cause iPPSDs, as these natural variants did not alter intrinsic GDP/GTP binding or conformational dynamics.

## 4. Materials and Methods

### 4.1. Materials

The protease inhibitor cocktail was purchased from BioVision (Mountain View, CA, USA). Deuterium oxide was purchased from Cambridge Isotope Laboratories (Tewksbury, MA, USA). Chelating sepharose resin for Ni-IDA purification was purchased from Cytiva (Uppsala, Sweden). EDTA, Tris, and NaH_2_PO_4_ were purchased from Duchefa Biochemie BV (Haarlem, The Netherlands). HEPES, IPTG, leupeptin, lysozyme, kanamycin, and TCEP were purchased from Goldbio (St. Louis, MO, USA). Acetonitrile, H_2_O, and MeOH were purchased from JT Baker (Phillipsburg, NJ, USA). DNase I was purchased from Roche (Basel, Switzerland). BODIPY-FL-GTP and BODIPY-FL-GTPγS were purchased from Thermo Fisher Scientific (Waltham, MA, USA). Acetic acid, benzamidine, formic acid, GDP, phosphoric acid, imidazole, and MgCl_2_ were purchased from Sigma–Aldrich (St. Louis, MO, USA).

### 4.2. Expression and Purification of WT and Mutant Gαs

Recombinant Gαs and Gαs mutants with an N-terminal 6×His-tag were cloned into pET28a. The mutant constructs were generated by site-directed mutagenesis using the polymerase chain reaction (PCR). The sequence of all constructs was confirmed by a DNA sequencing service (Bionics, Seoul, Korea).

Gαs WT and Gαs mutants were transformed into *Escherichia coli* LOBSTR (Kerafast, EC1002)-competent cells, and the transformed cells were grown in Terrific Broth containing antibiotics at 37 °C until the OD_600_ reached 0.6–0.8. Protein expression was induced using 0.03 mM IPTG, and the cells were further incubated at 16 °C for 24 h. For protein purification, the cell pellets were collected and resuspended in lysis buffer (20 mM Tris-HCl pH 7.4, 300 mM NaCl, 2 mM MgCl_2_, 20 µM GDP, 1 µg/mL protease inhibitor cocktail, 2.5 µg/mL leupeptin, 10 µg/mL benzamidine, and 100 µM TCEP). Next, we added 5 mg/mL lysozyme and incubated the mixture for 30 min at room temperature. The lysate was then incubated with 10 µg/mL DNase I for 30 min, and the supernatant was collected by centrifugation at 175,000× *g* for 25 min at 4 °C, supplemented with 20 mM imidazole, and loaded onto a Ni-IDA column equilibrated with lysis buffer. The Ni-IDA resin was thoroughly washed with wash buffer (20 mM HEPES, pH 7.4, 100 mM NaCl, 2 mM MgCl_2_, 20 µM GDP, 1 µg/mL protease inhibitor cocktail, 2.5 µg/mL leupeptin, 10 µg/mL benzamidine, 100 µM TCEP, and 20 mM imidazole). Proteins were eluted using an elution buffer (20 mM HEPES pH 7.4, 100 mM NaCl, 2 mM MgCl_2_, 20 µM GDP, 1 µg/mL protease inhibitor cocktail, 2.5 µg/mL leupeptin, 10 µg/mL benzamidine, 100 µM TCEP, and 250 mM imidazole). Further purification was performed using an ÄKTA FPLC and a Superdex-200 (10/300) column. Protein fractions were collected in elution buffer (20 mM HEPES pH 7.4, 100 mM NaCl, 2 mM MgCl_2_, 20 µM GDP, and 100 µM TCEP) by monitoring absorbance at 280 nm.

### 4.3. HDX-MS Analysis

All protein samples were prepared at a concentration of 60 µM. Gα protein (5 µL) was mixed with 25 µL of D_2_O buffer (20 mM HEPES pD 7.4, 100 mM NaCl, 2 mM MgCl_2_, 10 µM GDP, and 100 µM TCEP), and the samples were incubated for 10, 100, 1000, and 10,000 s on ice and quenched with 30 µL of ice-cold quench buffer (0.1 M NaH_2_PO_4_ pH 2.01, 20 mM TCEP, and 10% glycerol). The quenched samples were snap-frozen on dry ice and stored at −80 °C. Non-deuterated samples were prepared by mixing 5 µL of protein samples with 25 µL of their respective H_2_O buffers, followed by quenching and freezing as described above.

The quenched samples were digested and isolated using an HDX-UPLC-ESI-MS system (Waters, Milford, MA, USA). Briefly, the quenched samples were thawed and immediately passed through an immobilized pepsin column (2.1 × 30 mm) (Life Technologies, Carlsbad, CA, USA) at a flow rate of 100 µL/min in 0.05% formic acid in H_2_O at 12 °C. The peptic fragments were collected using a C18 VanGuard trap column (1.7 µm × 30 mm) (Waters) for desalting with 0.05% formic acid in H_2_O and then separated using an Acuity UPLC C18 column (1.7 µm, 1.0 × 100 mm) (Waters) at a flow rate of 40 µL/min with an acetonitrile gradient, with mobile phase B starting from 8% and increasing to 85% over 8.5 min. Mobile phase A was 0.15% formic acid in H_2_O and mobile phase B was 0.15% formic acid in acetonitrile. The pH of the buffers was adjusted to 2.5, and the system was maintained at 0.5 °C (except for pepsin digestion, which was performed at 12 °C) to minimize the back-exchange of deuterium. Mass spectral analyses were conducted using a Xevo G2 quadrupole time-of-flight (Q-TOF) instrument equipped with a standard ESI source in MS^E^ mode (Waters) in positive ion mode. The capillary, cone, and extraction cone voltages were set to 3, 40, and 4 V, respectively. The source and desolvation temperatures were set to 120 °C and 350 °C, respectively. The trap and transfer collision energies were set to 6 V, and the trap gas flow rate was set to 0.3 mL/min. Sodium iodide (2 µg/µL) was used to calibrate the mass spectrometer, and [Glu1]-Fibrinopeptide B (200 fg/µL) in MeOH:water (50:50 (*v*/*v*) + 1% acetic acid) was used for lock mass correction. The ions at a mass-to-charge ratio (*m*/*z*) of 785.8427 were monitored at a scan time of 0.1 s with a mass window of ± 0.5 Da. The reference internal calibrant was introduced at a flow rate of 20 µL/min, and all spectra were automatically corrected using lock mass. Two independent interleaved acquisition functions were created. The first function, typically set at 4 eV, collects low-energy or unfragmented data, whereas the second function collects high-energy or fragmented data, typically obtained using a collision ramp from 30 to 55 eV. Ar gas was used for collision-induced dissociation (CID). Mass spectra were acquired in the *m*/*z* range 100–2000 for 10 min. ProteinLynx Global Server 2.4 (Waters) was used to identify peptides from the non-deuterated samples with variable methionine oxidation modification and a peptide score of 6. DynamX 3.0 (Waters) was used to determine the level of deuterium uptake for each peptide by measuring the centroid of the isotopic distribution.

### 4.4. BODIPY-GTPγS Assay

Nucleotide-binding to Gα proteins was determined by measuring the change in the fluorescence intensity of BODIPY-FL-GTPγS in an imaging buffer (20 mM HEPES pH 7.4, 1 mM EDTA, 10 mM MgCl_2_, 100 µM TCEP, and 10 µM GDP). The fluorophore was excited at 485 nm (bandwidth, 14 nm), and the emission spectrum was recorded at 535 nm (bandwidth, 25 nm) using a TriStar2 S LB 942 Multimode Microplate Reader (Berthold Technologies, Bad Wildbad, Germany). The basal fluorescence of the imaging buffer was determined by measuring the fluorescence intensity without protein samples for 120 s. Gαs protein samples prepared in buffer (20 mM HEPES pH 7.4, 1 mM EDTA, 10 mM MgCl_2_, 10 µM GDP, and 100 µM TCEP) were mixed with the imaging buffer with or without 250 nM BODIPY-GTPγS (1:10 dilution; final Gα concentration: 0.79 µM). The change in fluorescence intensity was measured for 25 min at 1.2 s intervals in a 96-well black plate. All experiments were conducted at room temperature.

### 4.5. Statistical Analysis

The HDX-MS data are presented as the mean ± S.E.M of three independent experiments. Student’s *t*-test was used to evaluate differences in HDX-MS data. Differences were considered statistically significant at *p* < 0.05.

## Figures and Tables

**Table 1:** Natural variants reported in Gαs.

Natural Variants *	Loss-of-Function					Gain-of-Function	
	AHO ^a^	PHP1A ^b^	PHP1B ^c^	PHP1C ^d^	POH ^e^	MAS ^f^	Tumor
L99P	[20]						
I106S	[21]	[21]					
P115L	[22]						
D156N		[23]					
V159M		[23]					
R165C	[20]						
R201C						[17]	[24]
R201G						[25]	
R201H						[17,26]	[24]
R201S							[27]
R201L							[28]
Q227H							[29]
Q227R							[24]
R231H	[30]	[30,31,32]					
T242I	[33]						
F246S	[33]						
S250R	[34]						
R258W	[35]						
E259V	[33]						
R280G		[36]					
W281R					[37]		
K338N	[38]	[38]					
A366S		[39]					
I382del			[40]				
R385H	[41]						
L388R				[42]			
E392K				[42]			

* Colors indicate the residues shown in Figure 1A. ^a^ Albright hereditary osteodystrophy; ^b^ Pseudohypoparathyroidism 1A; ^c^ Pseudohypoparathyroidism 1B; ^d^ Pseudohypoparathyroidism 1C; ^e^ Progressive osseous heteroplasia; ^f^ McCune–Albright syndrome.

## Data Availability

The data presented in this study are available on request from the corresponding author.

## Supplementary Materials

The following supporting information can be downloaded at: https://www.mdpi.com/article/10.3390/ijms24044064/s1.
