# Supplementary material for: Structural and Functional Implication of Natural Variants of Gαs
Source: Int J Mol Sci. 2023 Feb 17;24(4):4064. doi: 10.3390/ijms24044064 (PMC9959179; doi:10.3390/ijms24044064)
Supplement: Supplementary file 1 [file ijms-24-04064-s001.zip › ijms-2194830-supplementary.pdf]

Figure S1. Peptic peitdes analyzed for HDX-MS

(A) WT vs. I106S

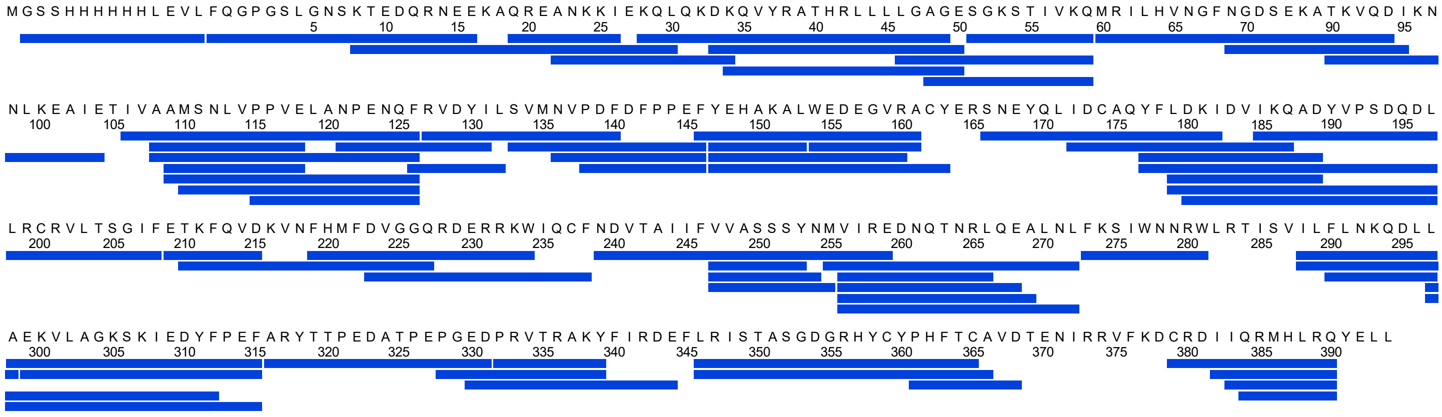

Total: 73 Peptides, 93.7% Coverage, 2.72 Redundancy

(B) WT vs. P115L or R165C

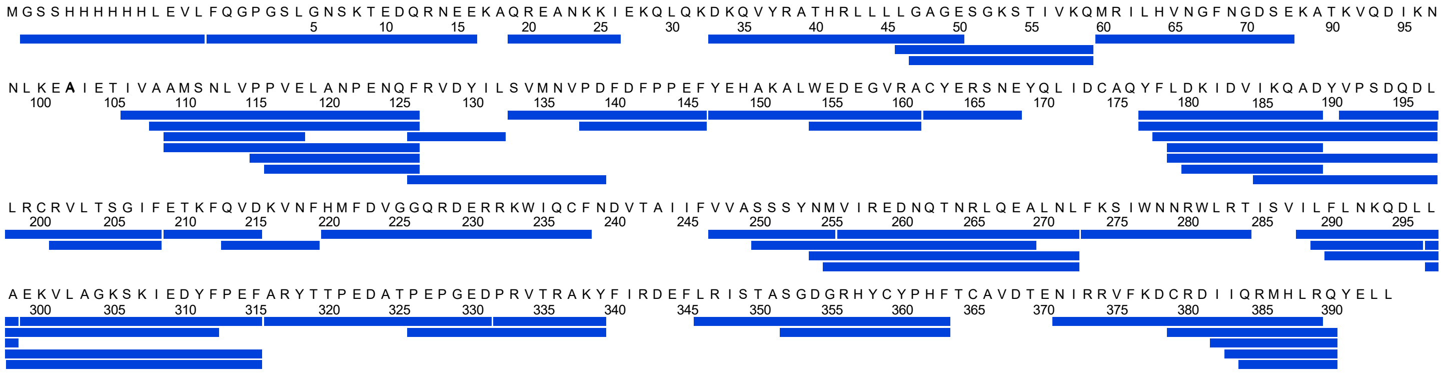

Total: 55 Peptides, 84.1% Coverage, 2.23 Redundancy

(C) WT vs. D156N or V159M

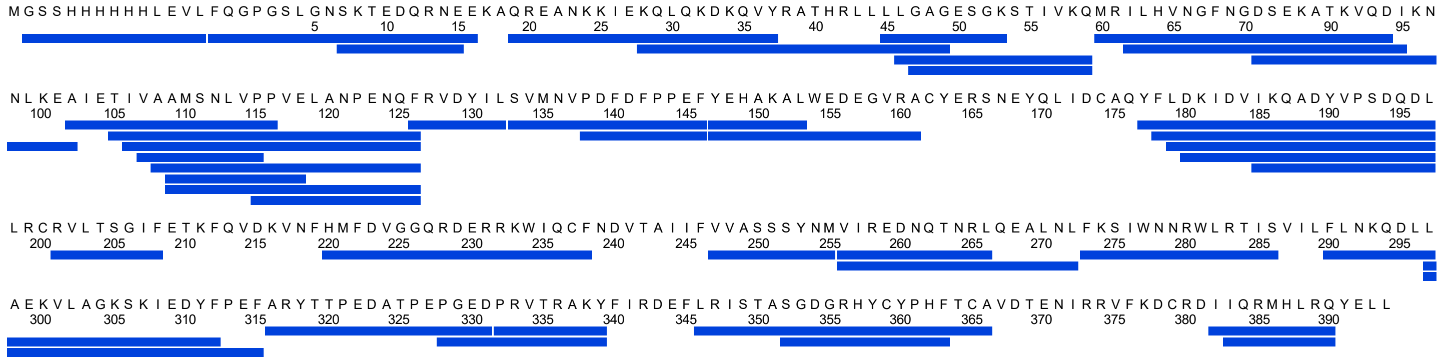

Total: 45 Peptides, 82.9% Coverage, 1.98 Redundancy

(D) WT vs. T242I

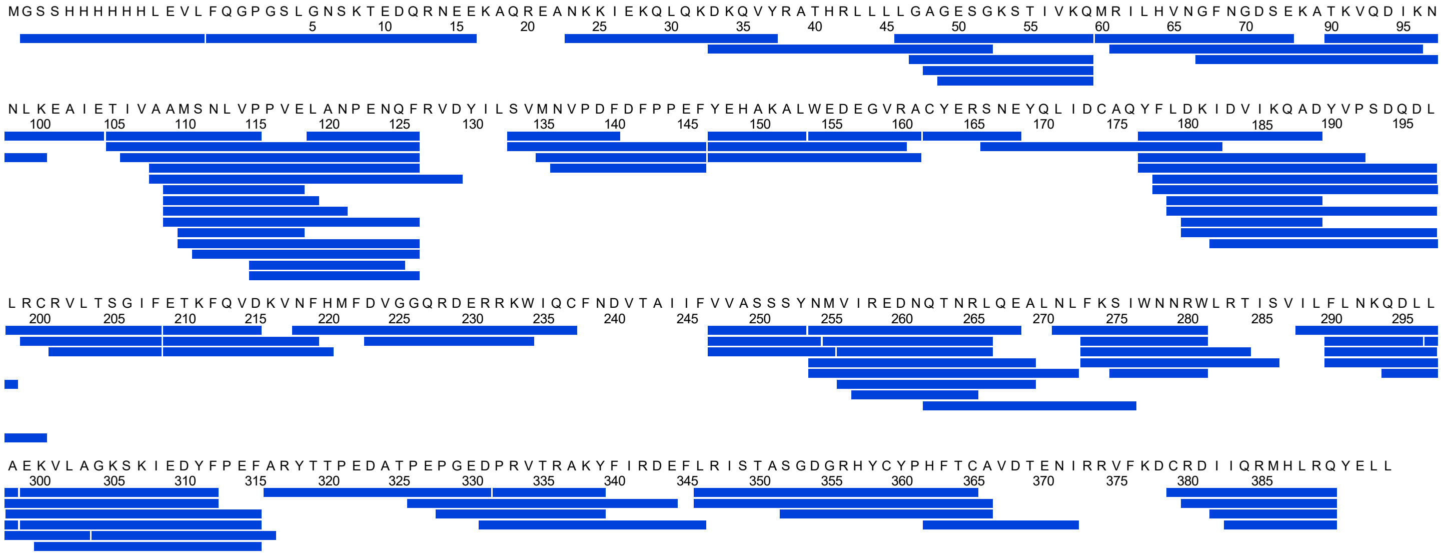

Total: 95 Peptides, 92.4% Coverage, 3.50 Redundancy

(E) WT vs. R280G or W281R

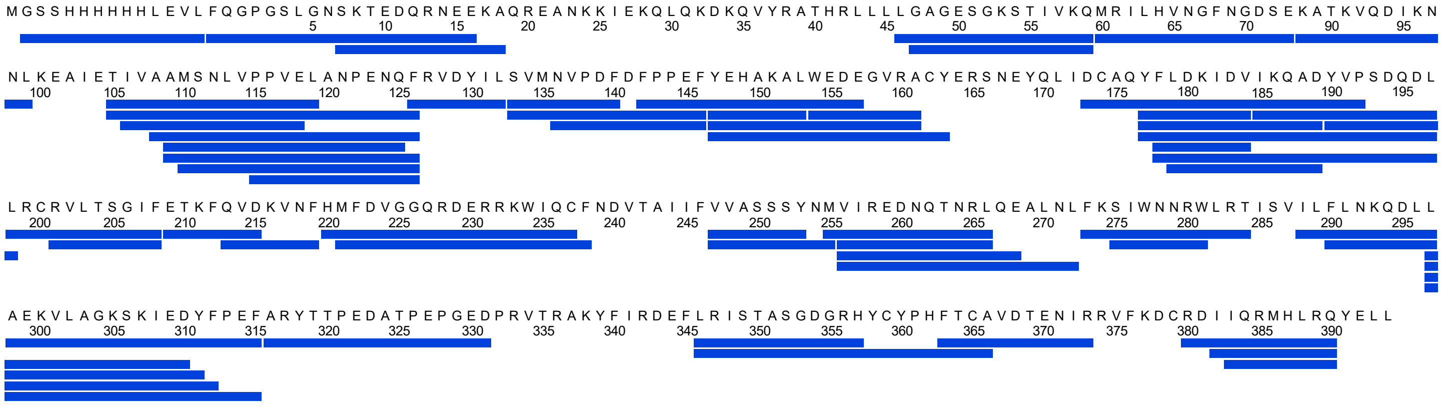

Total: 61 Peptides, 80.6% Coverage, 2.50 Redundancy
